# Encoding and Perception of Electro-communication Signals in *Apteronotus leptorhynchus*

**DOI:** 10.3389/fnint.2019.00039

**Published:** 2019-08-20

**Authors:** Michael G. Metzen

**Affiliations:** Department of Physiology, McGill University Montreal, Montreal, QC, Canada

**Keywords:** electro-communication, chirps, weakly electric fish, coding, perception

## Abstract

Animal communication plays an essential role in triggering diverse behaviors. It is believed in this regard that signal production by a sender and its perception by a receiver is co-evolving in order to have beneficial effects such as to ensure that conspecifics remain sensitive to these signals. However, in order to give appropriate responses to a communication signal, the receiver has to first detect and interpret it in a meaningful way. The detection of communication signals can be limited under some circumstances, for example when the signal is masked by the background noise in which it occurs (e.g., the cocktail-party problem). Moreover, some signals are very alike despite having different meanings making it hard to discriminate between them. How the central nervous system copes with these tasks and problems is a central question in systems neuroscience. Gymnotiform weakly electric fish pose an interesting system to answer these questions for various reasons: (1) they use a variety of communication signals called “chirps” during different behavioral encounters; (2) the central physiology of the electrosensory system is well known; and (3) most importantly, these fish give reliable behavioral responses to artificial stimuli that resemble natural communication signals, making it possible to uncover the neural mechanisms that lead to the observed behaviors.

## Introduction

The gymnotiform weakly electric fish,* Apteronotus leptorhynchus* uses active electroreception by means of a self-generated field (electric organ discharge, EOD) surrounding its body to navigate and communicate with conspecifics (Bennett, 1971; Zupanc et al., 2001). The EOD in this species can be described by a sinusoidal waveform of a specific frequency within the range of about 700–1,000 Hz. It has been shown that the individual EOD frequency is highly constant over long periods of time (i.e., hours), giving rise to a coefficient of variation of the EOD cycle period as low as 10^−4^. This makes the mechanism generating the EOD the most regular biological oscillator known (Moortgat et al., 1998). The meaningful stimulus for these fish is thus the modulations of their own EOD caused either by objects (electro-location) or during social interactions (electro-communication; MacIver et al., 2001; Kelly et al., 2008). Perturbations of the electric field due to objects or conspecifics are sensed by an array of cutaneous electroreceptors and are further processed downstream to finally elicit appropriate behaviors.

A specific type of electro-communication signal occurs when nearby fish briefly modulate their EOD frequencies. These signals are known as “chirps” and have been the focus of research for many years in terms of behavioral relevance (Hagedorn and Heiligenberg, 1985; Zupanc et al., 2006; Hupé and Lewis, 2008; Gama Salgado and Zupanc, 2011; Henninger et al., 2018) as well as their encoding across different stages of the central nervous system (Benda et al., 2005, 2006; Hupé et al., 2008; Marsat et al., 2009; Vonderschen and Chacron, 2009; Marsat and Maler, 2010; Metzen et al., 2016; Metzen and Chacron, 2017; Allen and Marsat, 2018). Because chirps can occur in different social settings, they must be reliably detected within complex backgrounds. No less important is the distinction of chirps in different situations within the same social encounter. As such, the production of chirps, as well as their perception and central processing by the members of the same species, must co-evolve in order to ensure that conspecifics remain sensitive and responsive to these signals (Allen and Marsat, 2019).

In the following, I will review the current advances about the central processing and perception of chirp signals in the weakly electric fish, *Apteronotus leptorhynchus*. I will first write about social communication signals in this species in general before briefly explaining the electrosensory pathway involved in signal processing. I will then give a brief overview about chirp encoding in different stages of sensory processing and finally give some insights on chirp production and chirp perception on the behavioral level.

## Social Communication Signals in *Apteronotus Leptorhynchus*

**Figure 1:**
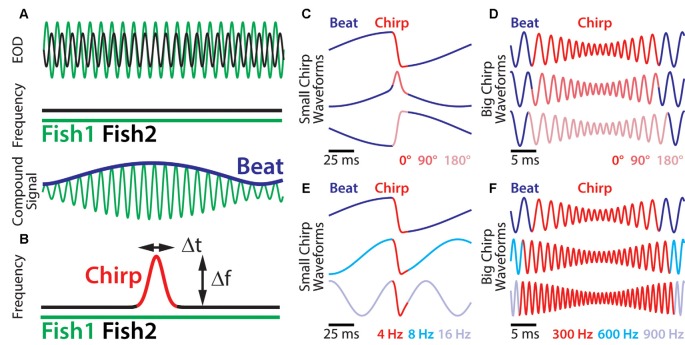
Small chirp stimuli are more heterogeneous than big chirps. **(A)** When two fish are in close proximity, their individual electric organ discharges (EODs; top green and black traces) create alternating regions of constructive and destructive interference. This interference results in a sinusoidal amplitude modulation (AM; i.e., a beat, bottom blue trace) of the summed signal (bottom green trace) that oscillates at the difference EOD frequency. **(B)** During a chirp (red), the emitter fish transiently increases its EOD frequency (black trace), while the receiver fish’s EOD frequency (top green trace) remains constant. A chirp can thus be characterized by its frequency increase and duration. **(C)** Resulting waveforms of small chirp stimuli (red) with fixed duration (14 ms) and frequency increase (60 Hz) within a 4 Hz beat (blue) occurring at different phases (dark red: 0°; light red: 90°; pink: 180°). **(D)** Resulting waveforms of big chirp stimuli (red) with fixed duration (25 ms), frequency increase (600 Hz) and amplitude drop (70%) within a 300 Hz beat (blue) occurring at different phases (dark red: 0°; light red: 90°; pink: 180°). **(E)** Resulting waveforms of small chirp stimuli (red) with fixed duration (14 ms) and frequency increase (60 Hz) occurring at the same beat phase (0°), but within different beat frequencies (dark blue: 4 Hz; cyan: 8 Hz; light blue: 16 Hz). **(F)** Resulting waveforms of big chirp stimuli (red) with fixed duration (25 ms) and frequency increase (600 Hz) occurring at the same beat phase (0°), but within different beat frequencies (dark blue: 300 Hz; cyan: 600 Hz; light blue: 900 Hz). Figures are adapted from Aumentado-Armstrong et al. (2015) and Metzen and Chacron (2017).

Social communication signals in *A. leptorhynchus* can be classified into different types, depending on the social context. Figure 1 describes different types of electrosensory stimuli and shows examples of stimulus waveforms associated with electro-communication signals under different conditions. The simplest signal in this regard occurs when two fish are in close proximity (<1 m). Then the interference of their EODs creates an amplitude modulation (AM) or beat that oscillates at the difference frequency (*dF*) between the two individual EOD frequencies (Figure 1A). Since *A. leptorhynchus* have been reported to display a sexual dimorphism in baseline EOD frequency (males tend to have higher EOD frequencies than females; Meyer et al., 1987), the *dF* contains important information about the sexual identity of a conspecific: same sex-encounters typically result in a low beat frequency (<50 Hz), whereas opposite-sex encounters result in higher beat frequencies (>50 Hz; Benda et al., 2006).

Although the EOD of *A. leptorhynchus* displays a high degree of constancy (Bullock, 1969), transient modulations of the frequency and/or amplitude occur spontaneously or during social interactions. A huge variety of EOD modulations have been described (Hagedorn and Heiligenberg, 1985; Engler and Zupanc, 2001). Some of these modulations are known as “chirps” and represent commutation signals that are actively generated by the fish during social interactions and different types of chirps have been identified (see Zakon et al., 2002). During a chirp event, one fish increases its EOD frequency for a short amount of time (Figure 1B). Although the behavioral meaning of chirps is still not entirely clear, two chirp types (type I, or “big chirps” and type II, or “small chirps”) have been the focus of extensive research on both, the behavioral level as well as on the encoding of them at several stages of sensory processing (Benda et al., 2005, 2006; Marsat et al., 2009; Marsat and Maler, 2010; Vonderschen and Chacron, 2011; Aumentado-Armstrong et al., 2015; Metzen et al., 2016; Metzen and Chacron, 2017; Allen and Marsat, 2018; Henninger et al., 2018).

A clear distinction between chirp types can be made based on two features: the increase in EOD frequency during a chirp and its duration (Figure 1B, red). While big chirps are characterized by large frequency increases (up to 1,000 Hz) that last between 20 and 30 ms, small chirps have smaller frequency increases (30–150 Hz) and shorter durations (10–18 ms). Big chirps are further accompanied by a significant drop in amplitude (up to 75%), whereas only negligible changes in amplitude (about 2%) have been reported for small chirps (Hagedorn and Heiligenberg, 1985; Zupanc and Maler, 1993; Bastian et al., 2001; Triefenbach and Zakon, 2003). Furthermore, big and small chirps occur at all phases of the beat with uniform probability (Aumentado-Armstrong et al., 2015). As such, chirp stimuli can display very heterogeneous waveforms (Zupanc and Maler, 1993; Benda et al., 2006). Especially for small chirps, the beat phase at which a chirp occurs can have huge effects on the resulting waveform (Figure 1C), whereas the waveforms of big chirps appear more self-similar across beat phases (Figure 1D; Aumentado-Armstrong et al., 2015). Similar effects on the chirp waveforms are obvious for different background beat frequencies (Figures 1E,F) and different combinations of frequency increase and duration most likely will impact the stimulus waveform as well.

Sociologically, big chirps occur most likely in behaviors associated with courtship contexts (Bastian et al., 2001; Engler and Zupanc, 2001) with more distant *dF*s (Zupanc et al., 2006; Hupé et al., 2008; Fugère and Krahe, 2010). In contrast, small chirps are commonly seen as aggressive intraspecific communication signals occurring on small *dFs* (Hagedorn and Heiligenberg, 1985; Bastian et al., 2001; Hupé and Lewis, 2008). It is well known that small chirps serve as a predictor of attacks during antagonistic encounters. As such, they are positively correlated with the overall expressed aggression of an animal, thus supporting the dominance hypothesis (Triefenbach and Zakon, 2008). In this regard, small chirps have been also shown to play an important role in mediating conspecific aggression (Hupé and Lewis, 2008). However, a recent field study showed that small chirps are also emitted during courtship behaviors between nearby fish of opposite sex (Henninger et al., 2018).

As for communication signals in other modalities and species (Allee et al., 2008, 2009; Gutzler et al., 2011; Beis et al., 2015; Wohr et al., 2015), processing of chirps by central neurons as well as chirping behavior has been shown to be regulated by serotonin (Deemyad et al., 2013; Larson et al., 2014) or steroid hormones (Dunlap et al., 2013; Smith, 2013).

## Electrosensory Pathway

Gymnotiform weakly electric fish possess a specialized electric organ whose discharges generate an oscillating electric field around the animal’s body. The electric organ is composed of so-called electrocytes. In members of the family Apteronotidae, the electrocytes are derived from motor axons, whereas the electric organ of all other weakly electric fish species is composed of derived muscle cells (Bennett, 1971). The synchronous activity, as well as the organization of the electrocytes within the electric organ, thus defines the EOD in terms of frequency and amplitude. Electrocytes receive command pulses from neurons located in the pacemaker nucleus in the medulla oblongata (Bennett, 1971), making the EOD frequency a direct consequence of the oscillation frequency of the pacemaker nucleus. A detailed description of the neural control of the electric organ can be found elsewhere (Bennett, 1971).

**Figure 2:**
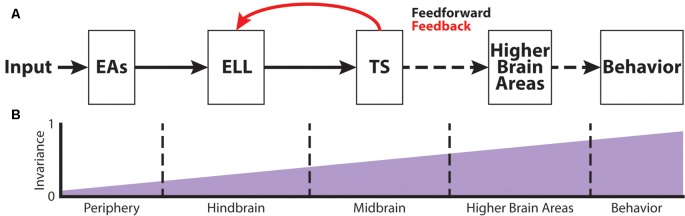
The phase invariance representation of small chirps increases across successive brain areas. **(A)** Schematic showing the different stages of sensory processing in the electrosensory system. **(B)** Schematic showing the increase in phase invariance across successive stages of electrosensory processing. Figures are modified from Metzen et al. (2016) and Metzen et al. (2018).

Figure 2A shows the feedforward electrosensory pathway across different stages of sensory processing, leading to behavior (black arrows), as well as an important feedback pathway (red). Perturbations of the electric field due to objects (i.e., electro-location) or the EODs of conspecifics (i.e., electro-communication) in the vicinity are sensed by peripheral P-type tuberous electroreceptors (electrosensory afferents, EAs; Bullock, 1969; Nelson et al., 1997; Figure 2A). Each EA trifurcates and projects topographically to three maps within the hindbrain electrosensory lateral line lobe (ELL): the centro-medial (CMS), centro-lateral (CLS) and lateral (LS) segments (Carr et al., 1982; Heiligenberg and Dye, 1982; Krahe and Maler, 2014). The ELL contains two main types of pyramidal cells (PCells): ON- and OFF-type cells (Clarke et al., 2015). ON-type cells respond to increases in EOD amplitude with increased spiking activity, whereas OFF-type cells respond with decreased spiking activity to increases in EOD amplitude. Based on physiological, morphological and molecular criteria, ON- and OFF-type PCells can be further subdivided into deep, intermediate and superficial cell types (Maler, 2009). The apical dendrites of superficial and intermediate PCells reach into the molecular layer and receive feedback signals from higher-order brain areas (Figure 2A). This feedback originates from the deep PCells. Furthermore, PCells are the sole output of the ELL and thus project to the torus semicircularis (TS), a midbrain nucleus. TS neurons project to higher brain areas such as the nucleus electrosensorius (nE), which projects to the prepacemaker nucleus (PPn). The PPn projects to the pacemaker nucleus (Pn), which then sends command signals to the electric organ, thereby completing the sensorimotor loop.

## Chirp Coding at the Sensory Periphery

Peripheral EAs respond to AMs of the fish’s own EOD with phase-locking (Hopkins, 1976; Bastian, 1981; Nelson et al., 1997). Hence, the probability of firing an action potential depends on the amplitude as well as the frequency of the AM (Nelson et al., 1997). As such, phase-locking tends to be greater for either higher amplitudes or higher frequencies, which is a direct consequence of their high-pass frequency tuning characteristics (Bastian, 1981; Xu et al., 1996; Chacron et al., 2005). Because EAs also display strong spike-frequency adaptation, their responses to low-frequency AMs are reduced (Benda et al., 2005). However, this adaptation can be overcome by a chirp stimulus, because chirps transiently increase the AM frequency, thus increasing EA responses (Benda et al., 2005).

The transient increase in frequency together with the resulting phase-reset leads to synchronous spiking activity in the EA population (Benda et al., 2006). This spiking synchronization can be categorized into two events: synchronous excitation in EAs due to small chirps that occur at a beat phase <180° (“+ chirps”), and synchronous inhibition in EAs due to small chirps that occur at a beat phase >180° (“− chirps”; Metzen et al., 2016). This correlated activity appears to be more similar for different patterns of small chirp waveforms than the firing rate modulations of single units, allowing for the emergence of an invariant representation of small chirps early in the nervous system (Metzen et al., 2016). However, due to their high-pass tuning properties, EAs also display a considerable amount of phase-locking to higher beat frequencies (Xu et al., 1996; Nelson et al., 1997; Chacron et al., 2005; Metzen and Chacron, 2017), which then decreases phase invariant coding by correlated afferent activity (Metzen and Chacron, 2017). Big chirps, in contrast, desynchronize EA responses, because of the large frequency increase as well as the significant drop in amplitude (Benda et al., 2006).

Because EAs are broadly tuned to stimuli associated with electro-location and -communication, studying the processing of electro-communication signals in weakly electric fish has some limitations: the electrosensory system is exposed to interferences among these two categories of signals (Benda et al., 2013). This is because electro-communication signals of low amplitude can be obscured by distortions of the electric field due to objects in its vicinity. However, it has been shown that the electrosensory system actually uses intrinsic stochastic resonance (i.e., neuronal noise) in order to enhance information processing for weak signals (Benda et al., 2013).

## Chirp Coding at the Level of the Hindbrain

EAs project to the ELL in the hindbrain of *A. leptorhynchus* (Figure 2A) where they trifurcate to the different ELL maps, LS, CLS, and CMS (Carr et al., 1982; Krahe and Maler, 2014). As mentioned earlier, each segment is composed of superficial, intermediate and deep PCells that respond with excitation (ON-type) or inhibition (OFF-type) to increasing AMs (Bastian and Nguyenkim, 2001). Chirp encoding in ELL is strongly affected by feedback input (Marsat and Maler, 2012). While superficial and intermediate PCells receive large amounts of feedback on their apical dendrites, deep PCells only receive minimal feedback, but rather serve as the source of these feedback projections (Bastian et al., 2002, 2004). Due to the feedback input as well as different tuning properties across PCells (Krahe et al., 2008), big and small chirps are not processed within the same maps. While big chirps are encoded by PCells of all maps, LS turns out to be the most sensitive map for processing sensory information related to small chirps (Metzner and Juranek, 1997; Marsat et al., 2009). Moreover, ON-type PCells have been shown to encode the presence of a small chirp with a stereotyped burst response due to the feedback input (Marsat et al., 2009). Although the presence of either big or small chirps can be reliably detected by ELL PCells, the discrimination of AM waveforms associated with small chirps with different attributes is difficult (Marsat and Maler, 2010). Furthermore, it has been shown that the encoding strategy of ELL PCells makes it difficult to discriminate between the different chirp waveforms of small chirps if they occur on a low-frequency beat (Marsat et al., 2009; Allen and Marsat, 2018). In contrast, if occurring on top of a high-frequency beat, both small and big chirps produce heterogeneous responses, and variations in the chirp waveform can be accurately discriminated (Marsat and Maler, 2010; Allen and Marsat, 2018). However, it has been shown that PCell responses to small chirps occurring at different phases of the beat are more invariant than the responses of single EAs (Figure 2B; Metzen et al., 2016). This is because ON-type PCells respond with similar excitation to “+ chirps,” whereas OFF-type PCells respond with similar excitation to “− chirps” (Metzen et al., 2016).

## Chirp Coding at the Level of the Midbrain

The target region of ELL PCells is the midbrain TS (Figure 2A). TS neurons receive direct excitatory synaptic input from ELL PCells (Carr and Maler, 1985; McGillivray et al., 2012). Although TS consists a large number of different neuron types (~50), they can be divided into dense and sparse coders based on their baseline firing rate and response properties to electrosensory stimuli (Chacron et al., 2011; Vonderschen and Chacron, 2011). Dense TS neurons respond to electrosensory stimulation similarly to ELL PCells. In contrast, sparse TS neurons respond selectively to preferred stimulus attributes and are mostly silent to other stimuli (Vonderschen and Chacron, 2011; Sproule et al., 2015). It is indeed these sparse coders that have been shown to have a higher degree of phase invariance compared to EAs and ELL PCells (Figure 2B) as they selectively respond to “+ chirps” as well as to “− chirps” with excitation but will not respond to the beat (Vonderschen and Chacron, 2011; Metzen et al., 2016). These neurons most likely correspond to previously characterized “ON-OFF” neurons that respond to both increase and decreases in the stimulus (Partridge et al., 1981; Rose and Call, 1993) because they receive balanced input from ON- and OFF-type ELL PCells (Aumentado-Armstrong et al., 2015). However, it is expected that further refinement of the observed phase invariance occurs in more downstream brain areas such as the nucleus electrosensorius in the diencephalon that receives direct input from TS (Carr et al., 1981). Both categories of TS neurons project to higher brain areas (Sproule et al., 2015). As such, the two categories of TS neurons could hold complementary functions within the processing of electro-communication signals: sparse neurons would simply detect the occurrence of a chirp, whereas dense neurons would instead transmit contextual information about the chirp identity (Metzen et al., 2016).

## Chirp Production and Perception

Behavioral responses to chirp stimuli have been mostly quantified through a behavioral paradigm in where the fish is restrained within a tube. There, it has been shown that chirp production in *A. leptorhynchus* decreases for increasing beat frequencies (Bastian et al., 2001; Engler and Zupanc, 2001) and that males respond with increased chirp production to increasing stimulus intensities (Zupanc and Maler, 1993; Engler and Zupanc, 2001). Furthermore, chirps naturally occur at all beat phases (Zupanc and Maler, 1993; Walz et al., 2013; Aumentado-Armstrong et al., 2015). Moreover, it has been shown that chirps generated by one individual follow those of another with a preferred latency of approximately 500–1,000 ms (Zupanc et al., 2006). These so-called “echo responses” can be also elicited by using artificial signals consisting of frequency modulations with different durations and thus can be used to study the neural bases of chirping behaviors under different experimental conditions (Gama Salgado and Zupanc, 2011; Metzen et al., 2016; Metzen and Chacron, 2017). Echo responses to small chirps have been shown to be similar if chirps where delivered at random beat phases on a low beat frequency resulting in a high degree of phase invariance on the organismal level (Figure 2B; Metzen et al., 2016), but phase invariant perception decreases for increasing beat frequencies (Metzen and Chacron, 2017). However, this phase invariant perception of small chirps indicates that *A. leptorhynchus* actually perceives different waveforms associated with small chirps as belonging to the same category if they occur on low beat frequencies. The decreased chirp detectability on the behavioral level at high beat frequencies is most likely due to increased phase-locking seen in EAs to higher background beat frequencies. This, in turn, synchronizes the responses of EAs irrespective of the chirp attributes, which is then decoded downstream.

Interestingly, in close proximity, fish tend to rather emit chirps instead of biting one another (Hupé and Lewis, 2008). It is therefore hypothesized that antagonistic chirps are primarily used to temporarily “blind” the opponent as they suppress electrosensory neural responses to other relevant stimuli (Zakon et al., 2002; Hupé and Lewis, 2008).

## Conclusion

Electro-communication in weakly electric fish has been the focus of research for many years. However, most of these studies were conducted under laboratory conditions where the fish was restrained in a chirp chamber. Thereby, a lot of knowledge has been gained regarding the central processing of chirps and behavioral responses to them. However, how related behaviors such as chirp production and perception is affected under more natural conditions and with interacting individuals is not well understood to date. More studies are needed to identify the underlying mechanisms as well as to link these with observable behaviors. Moreover, a recent study revealed that there are robust behavioral responses in stimulus regimes that have been not considered in electrophysiological studies so far (Henninger et al., 2018). The reason for this is mainly due to the fact that such stimuli mainly occur in freely behaving animals within their natural habitats. The entire stimulus ensemble these fish are exposed to in their natural environment has thus not been sufficiently characterized. More field studies are needed in order to fully understand the natural stimulus dynamics.

*Apteronotus leptorhynchus* is particularly well suited for studying sensory processing of and behavioral responses to electro-communication signals for various reasons. First, due to the neurogenic nature of its electric organ, the EOD persists after injecting the animal with curare-like drugs. This allows a preparation in which the animal is awake and behaving allowing a direct link of neuronal responses to chirp stimuli with behavioral responses. This is different in weakly electric fish species that possess a myogenic derived electric organ like *Eigenmannia* sp. because injection of curare-like drugs will basically silence the EOD due to inhibition of acetylcholine receptors at the neuromuscular junction (Hitschfeld et al., 2009). Second, *A. leptorhynchus* has been shown to give reliable behavioral responses (i.e., echo responses) during various social interactions (Zupanc, 2009). Furthermore, studying the limits of central processing and perception of electro-communication signals using for example highly unnatural chirp stimuli is feasible, as it is easy to elicit neuronal and behavioral responses to artificial chirp stimuli. Last, distinct chirp waveforms (or types) can be associated with different behaviors in *A. leptorhynchus*, whereas chirping in *Eigenmannia* sp. has been observed mainly in the context of reproduction (Zupanc and Bullock, 2005). However, since *Eigenmannia* sp. chirps contain both low- and high-frequency components that drive different types of electroreceptors, facilitating the study of parallel processing of different chirp attributes in *Eigenmannia*, but not in *Apteronotus* (Stöckl et al., 2014).

## Significance for the Field

The study of electro-communication signals (chirps) in terms of behavioral relevance as well as the central encoding mechanisms has a long history in weakly electric fish research. Scientists were able to characterize a huge variety of different stimulus waveforms associated with chirp signals of different kinds and discovered various behaviors related to chirps. Over the last years, studies uncovered more and more details about the behavioral circumstances in which different chirp types occur. Moreover, neurophysiological experiments revealed how single units as well as populations of neurons at different stages of sensory processing encode the stimulus waveforms associated with different chirp types and identities. The use of more naturalistic experimental settings in order to study behavioral and neuronal responses to chirps has become more important in recent years and led to a more fundamental understanding of how chirps are centrally processed and perceived on the organismal level.

## Author Contributions

The author confirms being the sole contributor of this work and has approved it for publication.

## Conflict of Interest Statement

The author declares that the research was conducted in the absence of any commercial or financial relationships that could be construed as a potential conflict of interest.

## Abbreviations

AM: amplitude modulation
dF: delta frequency
EAs: electrosensory afferents
ELL: electrosensory lateral line lobe
EOD: electric organ discharge
PCells: pyramidal cells
TS: torus semicircularis.
